# Elemental Zn and its Binding Protein Zinc-α2-Glycoprotein are Elevated in HPV-Positive Oropharyngeal Squamous Cell Carcinoma

**DOI:** 10.1038/s41598-019-53268-1

**Published:** 2019-11-18

**Authors:** Kate Poropatich, Tatjana Paunesku, Alia Zander, Brian Wray, Matthew Schipma, Prarthana Dalal, Mark Agulnik, Si Chen, Barry Lai, Olga Antipova, Evan Maxey, Koshonna Brown, Michael Beau Wanzer, Demirkan Gursel, Hanli Fan, Alfred Rademaker, Gayle E. Woloschak, Bharat B. Mittal

**Affiliations:** 1 Department of Pathology, Northwestern University Feinberg School of Medicine, Chicago, IL 60611 USA; 2 Robert H Lurie Comprehensive Cancer Center, Northwestern University Feinberg School of Medicine, Chicago, IL USA; 3 Department of Radiation Oncology, Northwestern University Feinberg School of Medicine, Chicago, IL USA; 4 Center for Genetic Medicine, Northwestern University Feinberg School of Medicine, Chicago, IL USA; 5 Division of Hematology and Oncology, Northwestern University Feinberg School of Medicine, Chicago, IL USA; 6 X-ray Science Division, Advanced Photon Source, Argonne National Laboratory, 9700 South Cass Avenue, Argonne, IL 60439 USA; 7 Northwestern University Pathology Core Facility, Robert H Lurie Comprehensive Cancer Center, Northwestern University Feinberg School of Medicine, Chicago, IL USA; 8 Department of Preventive Medicine, Northwestern University Feinberg School of Medicine, Chicago, IL USA

**Keywords:** Head and neck cancer, Head and neck cancer, Prognostic markers, Prognostic markers

## Abstract

Human papillomavirus (HPV)-positive head and neck squamous cell carcinoma (HNSCC) is biologically distinct from HPV-negative HNSCC. Outside of HPV-status, few tumor-intrinsic variables have been identified that correlate to improved survival. As part of exploratory analysis into the trace elemental composition of oropharyngeal squamous cell carcinoma (OPSCC), we performed elemental quanitification by X-ray fluorescence microscopy (XFM) on a small cohort (n = 32) of patients with HPV-positive and -negative OPSCC and identified in HPV-positive cases increased zinc (Zn) concentrations in tumor tissue relative to normal tissue. Subsequent immunohistochemistry of six Zn-binding proteins—zinc-α2-glycoprotein (AZGP1), Lipocalin-1, Albumin, S100A7, S100A8 and S100A9—revealed that only AZGP1 expression significantly correlated to HPV-status (p < 0.001) and was also increased in tumor relative to normal tissue from HPV-positive OPSCC tumor samples. AZGP1 protein expression in our cohort significantly correlated to a prolonged recurrence-free survival (p = 0.029), similar to HNSCC cases from the TCGA (n = 499), where highest *AZGP1* mRNA levels correlated to improved overall survival (p = 0.023). By showing for the first time that HPV-positive OPSCC patients have increased intratumoral Zn levels and AZGP1 expression, we identify possible positive prognostic biomarkers in HNSCC as well as possible mechanisms of increased sensitivity to chemoradiation in HPV-positive OPSCC.

## Introduction

Human papillomavirus (HPV)-positive oropharyngeal squamous cell carcinoma (OPSCC) is biologically and clinically distinct from HPV-negative OPSCC and HPV status is a well-established favorable prognostic variable^1^. The continuously rising number of patients presenting with HPV-positive OPSCC in the United States has eclipsed the number of HPV-negative OPSCC^1–3^. Increased evidence demonstrates that even within HPV-positive head and neck squamous cell carcinoma (HNSCC), there are divergent clinical outcomes based on factors such as patient smoking history^4^. Immune-related tumor-extrinsic variables have been identified in HPV-positive OPSCC, including some by our group^5,6^, such as the phenotypic and morphologic features of tumor infiltrating lymphocytes. With regards to tumor-intrinsic variables, sex hormone overexpression and gene silencing through promoter methylation^7,8^ have recently been correlated to improved survival in HPV-positive OPSCC.

As cofactors for multiple enzymes and proteins, essential trace elements—iron (Fe), copper (Cu), cobalt (Co), manganese (Mn) and zinc (Zn)—are required for normal biological functions^9^. Elevated levels of some elements such as Fe, Cu and Zn have also been detected in the serum and tumor tissue from patients with different types of cancers^10–14^. At the same time, ample evidence indicates that different types of aggressive hormonally-driven human cancers (i.e. breast, prostate and ovarian) have low intratumoral Zn levels relative to normal tissue^15–17^. Zn exists largely bound to proteins and one type of Zn-binding proteins are metallothioneins, which are involved in Zn metabolism and have been implicated in chemoradiosensitivity and restoration of mutant p53 in tumor cells^18–23^. The analysis of trace elements in HPV-positive OPSCC may assist in better understanding their intrinsic enhanced sensitivity to chemoradiation.

In this study, we utilize elemental mapping of a small cohort of clinically-matched HPV-positive and negative OPSCC cases (n = 32) by X-ray fluorescence microscopy (XFM) and demonstrate Zn is increased in HPV-positive OPSCC tumor tissue relative to matched normal squamous mucosa. By performing a targeted analysis of the expression of six Zn-binding proteins—Lipocalin-1, zinc-α2-glycoprotein (AZGP1), albumin, S100A7, S100A8 and S100A9—in tumors from OPSCC patients (n = 68), we reveal AZGP1 is selectively overexpressed in a subpopulation of HPV-positive OPSCC patients and that its expression is an independent predictor of patient survival in this patient population.

## Results

### Patient population

A total of 75 cases of OPSCC with sufficient tissue for further testing were identified (Supplemental Fig. 1), of which 48 were HPV-positive. The majority of patients were male and the median age at the time of diagnosis was 58 years (Supplemental Table 1). While HPV-positive patients were more likely to be never-smokers compared to HPV-negative patients, this was not significant. Among different treatment modalities, combination surgery and chemoradiation was the most common, accounting for approximately half of all the patients in the cohort (Supplemental Table 1). As expected, HPV-negative OPSCC patients were more likely to have a recurrence, accounting for 59% of all recurrences (10/17), and the recurrence free survival (RFS) was significantly longer in HPV-positive patients compared to HPV-negative patients (3.71 years vs. 2.51 years, p = 0.001).

### Elemental measurement and mapping by XFM

**Figure 1:**
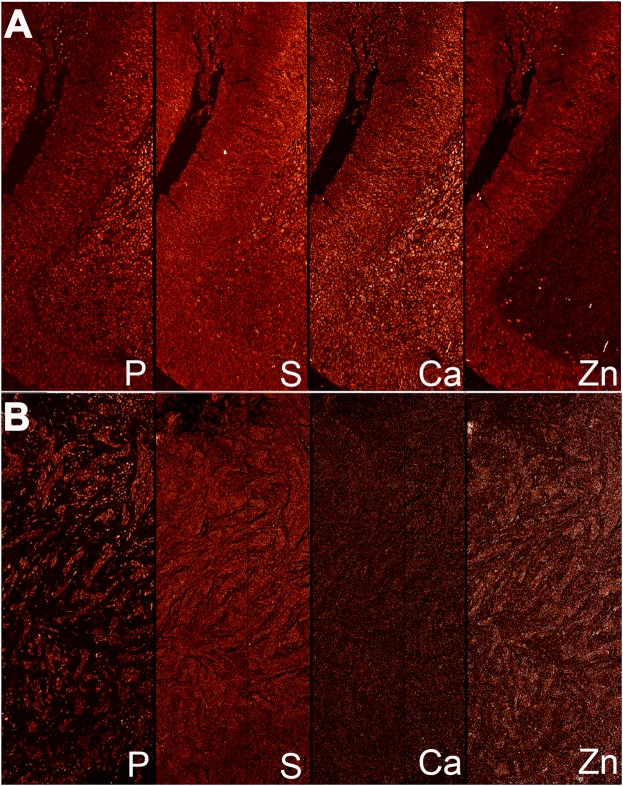
XFM elemental maps for a matched OPSCC patient pair. Medium resolution (beam focused to 0.5 μm spot) images were collected from formalin-fixed whole-tissue sections of tumor from patients with OPSCC (n = 32, 16 HPV-positive, 16 HPV-negative). Shown are the elemental maps for P, S (reference element for normalization), Fe, Ca and Zn in the tumor from a matched HPV-positive OPSCC (**A**) and HPV-negative OPSCC (**B**) patient pair.

**Figure 2:**
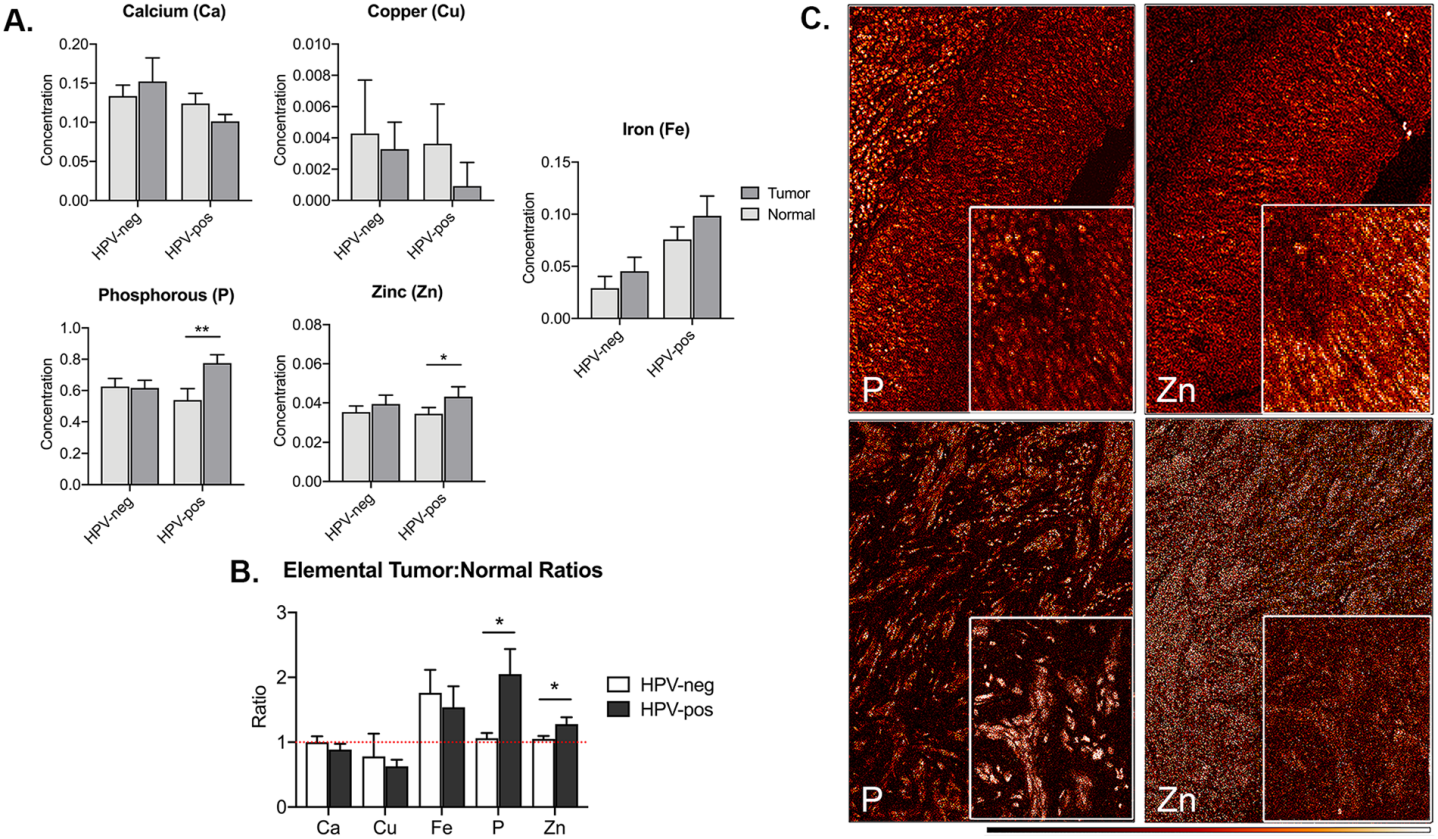
Quantified elemental concentrations in HPV-positive and -negative OPSCC cases. (**A**) Elemental concentrations for Ca, Cu, Fe, P and Zn in tumor and adjacent normal mucosa from OPSCC patients (n = 32). Data from scans were calibrated and per pixel counts were converted to elemental concentrations (μg/cm^2^) (Methods). Final measurement of elemental concentrations were normalized relative to sulfur. (**B**) Ratios of tumor-to-normal for individual samples were calculated for Ca, Cu, Fe, P and Zn for 16 HPV-positive and HPV-negative patient pairs (Methods). (**C**) Medium resolution XFM tumor elemental maps for P and Zn in a clinically-matched HPV-positive (top) and HPV-negative (bottom) OPSCC patient pair. These detailed maps are representative of larger tumor region scans shown in Fig. 1. For elemental quantification from this patient pair, see Supplemental Table 2. Bar graph data are mean ± s.e.m; statistical significance evaluated by a paired Student’s T test (a, b).  *P < 0.05; **P < 0.01.

Among the 16 elements mapped by low-resolution XFM scanning of tumor and histologically normal adjacent mucosa in OPSCC cases (n = 32), the quality of data was high and suitable for analysis for the following eight elements: P, S, K, Cl, Ca, Fe, Cu and Zn. Given that K and Cl measurements are often inaccurate in formalin-fixed paraffin-embedded (FFPE) samples^24^, we focused on Ca, Cu, Fe, P and Zn in HPV-positive and –negative patient pairs matched for different clinical features (Methods), using S to normalize the data (Methods, Fig. 1). Among these elements, normalized P and Zn

concentrations were significantly elevated in tumor tissue relative to matched adjacent normal mucosa from HPV-positive but not HPV-negative OPSCC cases (p = 0.0015 and 0.0228, respectively) (Fig. 2a). We then compared elemental tumor-to-normal ratios for 16 HPV-positive/HPV-negative patient pairs. Whereas Fe tumor:normal ratios were >1 for both HPV-positive and -negative OPSCC cases, only HPV-positive patients had ratios >1 for both P (p = 0.0263) and Zn (p = 0.0204) in the tumor tissue relative to normal mucosa (Fig. 2b). As differences in P concentrations may be in part ascribed to factors such as the increased nuclear content of HPV-positive tumors and aneuploidy nature of the tumors, which can increase DNA content^25^, we decided to focus our investigation on differences in Zn content between the two groups, especially in light of the fact that Zn has been shown to synergize with chemoradiation to induce sensitivity in different human tumor models^19–21,23^.

In order to perform a more in-depth analysis of elemental differences between HPV-positive and -negative samples, we scanned one of the matched HPV-positive and -negative patient pairs with medium-resolution XFM (Fig. 2c). Total quantities of elements (picograms) were determined from a region of interest (ROI) of 40 tumor cells per case (Supplemental Fig. 2, Supplemental Table 2). In this evaluation, the HPV-positive patient had approximately twice as high a quantity of Zn (1.67 picograms) compared to the HPV-negative patient (0.902 picogram).

### Identification of Zn-binding proteins by mass spectrometry

FFPE tumor tissues from an HPV-positive and -negative OPSCC patient pair were subjected to exploratory proteomic analysis. Among 276 proteins identified from laser-captured microdissection of tumor tissue, 47 unique proteins were present in the tumor from the HPV-positive patient, 36 were present in the tumor from the HPV-negative patient and the remaining 193 proteins were shared amongst both patient tumor samples (Supplemental Table 3). Among the most frequently detected proteins in both tumor samples were proteins indicative of epithelial tumor cell origin, including type I/type II keratins and actin.

As Zn had been identified from our XFM data as an element increased in the tumor from HPV-positive OPSCC patients, we further interrogated the 276 proteins for those that were Zn-binding in the tumor samples from either patient (Supplemental Table 3). Of these nine Zn-binding proteins, six were found in both tumor samples (AZGP1, Serum albumin, S100A8/Calgranulin-A, S100A9/Calgranulin-B, S100A7/Psoriasin, and Ubiquitin-40S ribosomal protein S27a), one was found only in the HPV-positive tumor sample (Lipocalin-1) and two were found in the HPV-negative tumor sample (Collagen alpha-1(XVIII) chain and Carbonic anhydrase 1).

### Zn-binding protein AZGP1 is elevated in subset of HPV-positive OPSCC cases

**Figure 3:**
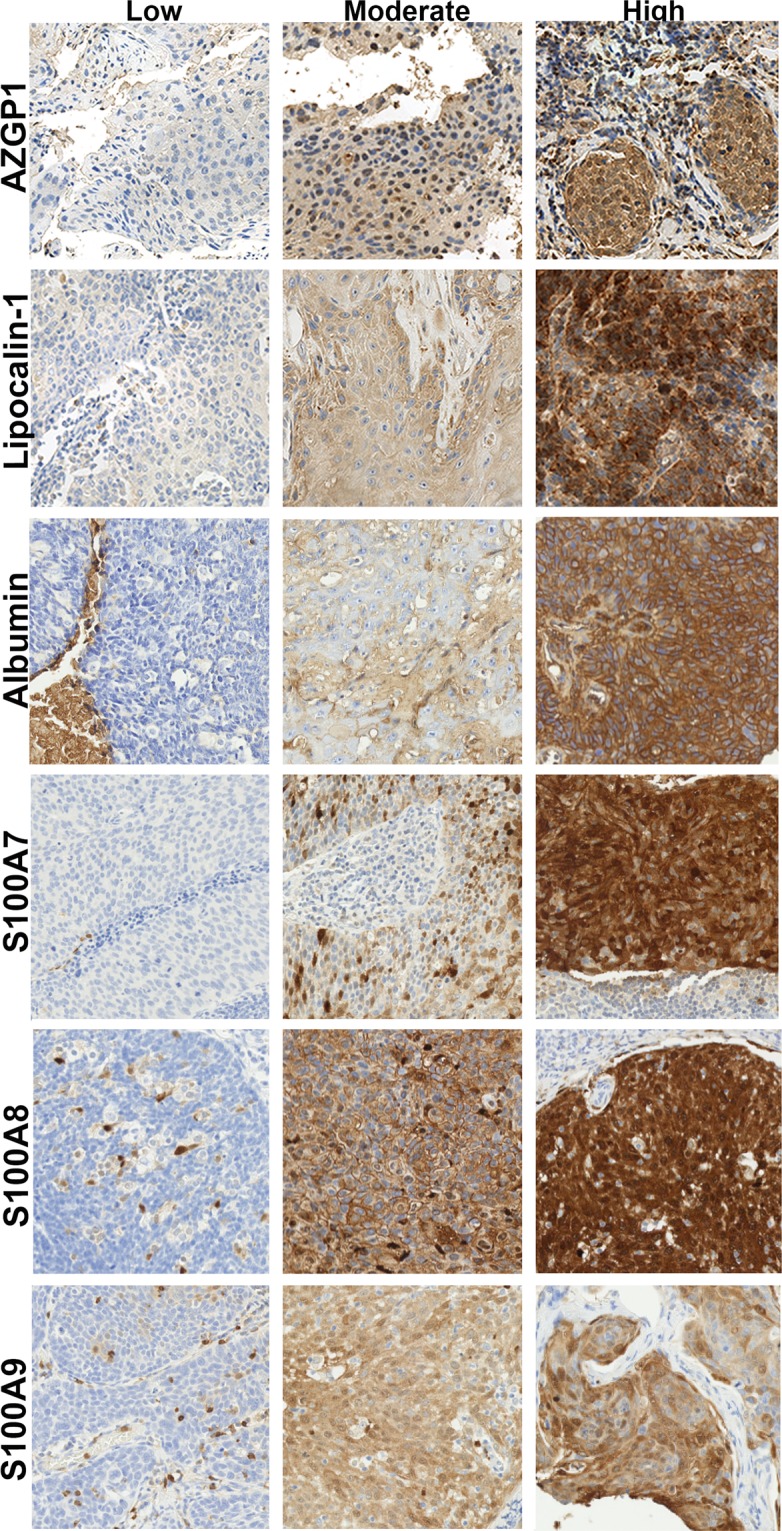
Zn-binding protein immunohistochemical staining in OPSCC. Examples from nanozoomer digitized scans (20X magnification) of low, moderate and high cytoplasmic staining of OPSCC tumor tissue for the Zn-binding proteins AZGP1, Lipocalin-1, serum Albumin, S100A7, S100A8 and S100A9.

We measured the expression of six different Zn-binding proteins in tissue microarrays (TMAs) from OPSCC patients by IHC in matched tumor and normal tissue, including: Lipocalin-1 (n = 63), AZGP1 (n = 68), albumin (n = 26), S100A7 (n = 53), S100A7 (n = 53), S100A8 (n = 51) and S100A9 (n = 50). We used a graded scoring system for staining intensity (low = 1, moderate = 2 and high = 3) in tumor and matched normal squamous mucosa. Among the six Zn-binding proteins, AZGP1 was the only protein with significant differential expression levels amongst HPV-positive and HPV-negative samples, with higher median expression in tumor relative to normal in HPV-positive cases compared to HPV-negative cases (Fig. 3, Supplemental Table 4). An opposite pattern was evident for HPV-negative cases in this cohort—tumor AZGP1 staining was reduced relative to adjacent normal mucosa. In contrast, median Lipocalin-1 staining was actually higher in the tumor of HPV-negative cases versus HPV-positive cases, albeit not statistically significant.

**Figure 4:**
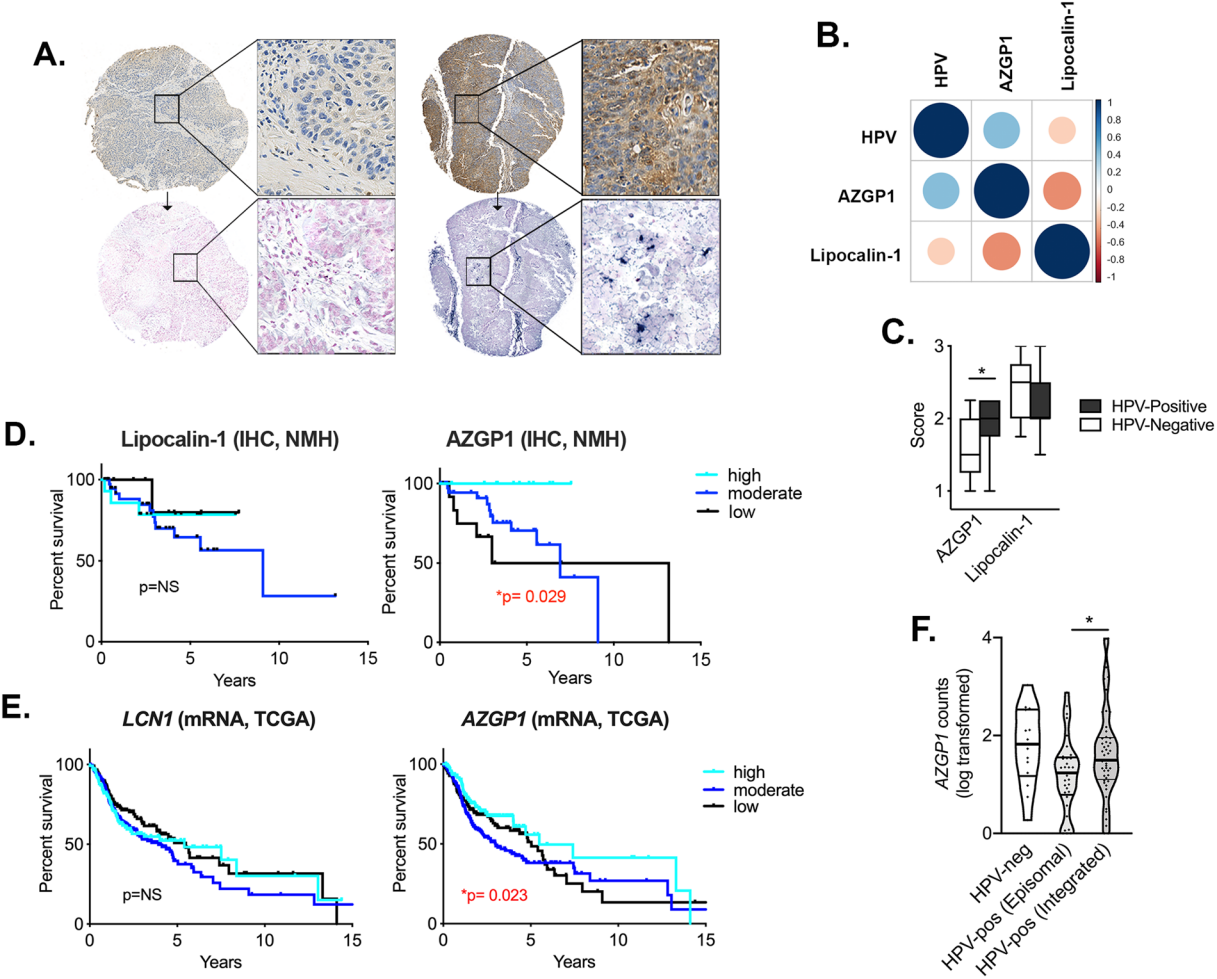
AZGP1 correlates to HPV-status and improved survival in OPSCC and HNSCC. (**A**) Comparison of AZGP1 immunohistochemical staining with HPV-status confirmed by in situ hybridization staining in HNSCC; Strong AZGP1 expression is detected in HPV-positive tumor tissue (right) and absent in HPV-negative tumor tissue (left) (**B**,**C**) A correlation matrix for HPV, AZGP1 and Lipocalin-1 in which HPV status significantly positively correlated to AZGP1 protein expression. Pearson correlation analysis. Box-and-whiskers plot shows comparison of HPV-positive and –negative OPSCC staining levels (low = 1, moderate = 2, high = 3) for AZGP1 and Lipocalin-1. Student’s T test. (**D**) Kaplan-Meier analysis of recurrence-free survival (RFS) in patients with low, moderate and high IHC levels for AZGP1 (n = 66) and Lipocalin-1 (n = 63). Overexpression of AZGP1 protein in OPSCC corresponded to significantly prolonged RFS (log-rank, p = 0.029). (**E**) Survival analysis in HNSCC patients (n = 499) from the TCGA with *LCN1* and *AZGP1* mRNA levels stratified as high, moderate and low. *AZGP1*-high cases have a significantly longer overall survival (log-rank, p = 0.023). (**F**) RNA-seq analysis of OPSCC cases from TCGA and GEO comparing *AZGP1* mRNA levels in HPV-negative, HPV-positive (integrated) and HPV-positive (episomal) OPSCC patients. Mean *AZGP1* mRNA counts for the HPV-integrated OPSCC cases were higher than in HPV-positive OPSCC with episomal HPV (p = 0.04637); Wilcoxon rank test. Data is shown as a violin plot (bold line at median and thin lines at upper and lower quartiles). *P < 0.05.

Correlation analysis revealed that HPV status significantly positively correlated to AZGP1 (p = 0.0001) but not Lipocalin-1 staining intensities; 13 of 14 (93%) of AZGP1-high cases were HPV-positive and among AZGP1-low cases (n = 15), two-third (n = 10) were HPV-negative (Fig. 4a–c). Additionally, Lipocalin-1 was significantly inversely correlated to AZGP1 expression (p = 0.001). Collectively, our findings suggest that in HPV-positive OPSCC, AZGP1 is overexpressed in a subset—approximately one-in-three—and that its protein expression may be downregulated in HPV-negative OPSCC patients.

### Patient survival is positively correlated to AZGP1 expression

 We chose to further focus on Lipocalin-1 and AZGP1 protein expression for patient survival analysis based on our findings of inverse expression trends in our patient cohort. As AZGP1 has been demonstrated to have anti-tumor activity and improved clinical outcome in cancer patients, we hypothesized its overexpression may portend improved patient survival [26,27]. Among our cohort cases with available survival data (Supplemental Table 1), we found that overexpression of AZGP1 significantly correlated to a longer RFS and that reduced expression correlated to a shorter RFS (p = 0.029); there were no recurrences in the AZGP-1 high group compared to nearly one-in-two recurrences in the AZGP1-low group, respectively (Table 1, Fig. 4d). In contrast, no survival benefit was present in our cohort of OPSCC patients based on Lipocalin-1 expression patterns.

**Table 1:** Uni- and Multivariate Survival Analysis of AZGP1 and Lipocalin-1 in OPSCC cohort.

Kaplan-Meier Estimate				AFT Model									
				Univariate		Multivariate							
						*HPV-status*		*T stage*^a^		*Smoking-status*^b^		*Treatment*^c^	
Protein	No.Patients (No.Events)	Survival Proportion	Log-rank *P* value	TR (95% CI)	*P*	TR (95% CI)	*P*	TR (95% CI)	*P*	TR (95% CI)	*P*	TR (95% CI)	*P*
AZGP1	66 (16) High = 12 (0) Medium = 39 (10) Low = 15 (6)	100 61.72 50.0	**0.029**	2.24 (2.20,2.28)	**<0.001**	1.22 (0.29,5.05)	NS	2.50 (0.90,6.93)	0.078	2.32 (2.26,2.38)	**<0.001**	**2.879 (2.85,2.91)**	**<0.001**
Lipocalin-1	63 (16) High = 15 (3)	78.57	NS	0.78 (0.15,4.02)	NS	1.38 (0.33,5.85)	NS	0.740 (0.099,5.54)	NS	0.77 (0.13, 4.52)	NS	0.897 (0.07,10.8)	NS
	Medium = 40 (12)	27.13											
	Low = 8 (1)	9											
		80.0											

Abbreviations: AFT = accelerated failure time, TR = time ratio, CI = confidence interval.

^a^Defined as T1 (reference) versus T2 or T3/T4. Method = Nelder-Mead and control = list(fnscale = 2500).

^b^Defined as never-smoker versus any smoking history.

^c^Defined as surgery with chemoradiation versus chemoradiation-only or surgery-only.

In order to determine whether the improved outcome of AZGP1 expression was dependent on other variables, covariates were addressed using the accelerated failure time (AFT) model (Table 1). Due to the fact that AZGP1 expression was almost exclusively in HPV-positive OPSCC, HPV status could not be used as a covariate in multivariate analysis. When adjusting for covariates, however, such as smoking status or treatment plan, AZGP1 protein expression positively correlated to improved patient survival.

When survival analysis was extended to The Cancer Genome Atlas (TCGA) to interrogate the relationship of *AZGP1* and *LCN1* mRNA transcript levels with HNSCC patient survival (n = 499), similar patterns were evident for both genes with their protein counterparts (Fig. 4e). Overexpression of *AZGP1* mRNA (n = 125, based on top quartile expression) correlated to longer overall survival in HNSCC patients, although low *AZGP1* mRNA levels (n = 124, based on bottom quartile expression) did not portend a worse overall outcome. No significant difference in overall survival was present based on *LCN1* expression levels.

### *AZGP1* mRNA levels correlate to HPV-integration status

Recent work has characterized the HPV-host status (integrated vs. episomal) in HNSCC, including OPSCC, from RNA-sequencing (RNA-seq) data in TCGA and Gene Expression Omnibus (GEO)^28^. We performed an analysis of *AZGP1* mRNA transcript levels from RNA-seq data available in the databases for HPV-positive and HPV-negative OPSCC cases, including a total of 97 patients with OPSCC from TCGA (n = 80) and GEO portals (n = 17)^28^. The majority of cases were HPV-positive (n = 83) compared to HPV-negative. While total mean levels were higher in the HPV-negative OPSCC cases compared to HPV-positive OPSCC cases, this was not statistically significant by ANOVA analysis (F value: 2.643, p = 0.0764). Among the HPV-positive OPSCC cases, approximately two-thirds had integrated HPV (n = 50) and one-third had episomal HPV (n = 33). HPV-integrated OPSCC cases had significantly higher levels of *AZGP1* mRNA transcripts compared to OPSCC cases with episomal HPV (Fig. 4f, p = 0.046, Wilcoxon rank test) (Fig. 4f).

## Discussion

The elemental and protein-based analysis of OPSCC patients presented here is a coordinated strategy to develop new understandings of the pathogenesis of OPSCC. This is the first attempt to characterize the composition of OPSCC tumor tissue by trace elements using XFM and to demonstrate that Zn-binding protein AZGP1 is a potential biomarker for positive prognosis in HNSCC. Our findings add to other work that also demonstrates that patient outcome in HPV-positive OPSCC is affected by variables such as smoking status, hormonal status, gene expression and methylation patterns and HPV integration status^4,7,8,28^.

Studies utilizing XFM have shown distinct patterns in the tumor microenvironment (TME) of different human cancers^29,30^. In this study we found that the normalized tumor-to-normal P and Zn ratios were elevated in HPV-positive OPSCC patients compared to the ratios for HPV-negative patients. In different aggressive human solid tumor models (i.e. breast, prostate and ovarian), intratumoral Zn levels are low relative to normal tissue^15–17^. As an antioxidant, Zn has been studied as a tool to interrupt multiple carcinogenesis-related pathways^31^ and administration of exogenous Zn has been implicated as a strategy to increase chemoradiosensitivity^18–23^. In HNSCC, the reduction of systemic Zn levels is correlated to aggressive tumor development—a pilot human longitudinal study of patients with advanced HNSCC revealed that in the immediate timeframe before cancer-related death, Zn levels drop from baseline in the majority of patients^32^. Subsequently, clinical trials of oral HNSCC have incorporated oral Zn therapy into their protocols as adjuvant therapy^33^.

Zn stabilizes the structurally complex DNA-binding domain of the tumor suppressor gene p53 and has also been shown to rescue wild-type p53 activity in mutant p53 by re-establishing chemosensitivity in p53-mutated cell lines^21^. While entirely speculative, our finding of increased intratumoral Zn content in HPV-positive OPSCC cases could provide justification for the intrinsic chemoradiosensitivity of this patient population and offer insight into ways to improve the clinical outcome of patients with HPV-negative HNSCC, who frequently harbor mutant p53.

Unlike P, which is predominantly associated with nucleic acids, the vast majority of intra- and extracellular Zn is bound to proteins, serving either structural or catalytic roles, collectively referred to as the ‘zinc proteome’^34^. While we could not conduct a complete survey of all possible Zn binding proteins, we tested for the expression of six Zn-binding proteins in our OPSCC cohort that we initially detected in OPSCC by mass spectroscopy—Lipocalin-1, AZGP1, albumin, S100A7, S100A8 and S100A9—and we found that only AZGP1 expression was significantly higher in HPV-positive OPSCC tumor cells.

AZGP1 is a soluble 40-kDa protein and major histocompatibility complex homolog and adipokine located on human chromosome 7q22.1. It is secreted by epithelial ductal cells of the salivary glands, breast, prostate and gastrointestinal tract^35,36^. In oral and buccal squamous epithelium, AZGP1 is intracellularly expressed in the mature layers of the stratified squamous epithelium^37^. AZGP1 expression may be related to HPV infection of human squamous epithelium—transcriptomic analysis of mature keratonicytes infected with HPV-16 demonstrated that AZGP1 mRNA is one of the six most-upregulated genes^38^. AZGP1 has one strong Zn-binding site and up to 15 weak Zn-binding sites. The strong Zn-binding site is located close to the lipid-binding helical groove that AZGP1 uses as Zn-binding ligands^39^. Zn, but no other divalent metals, induces the oligomerization of AZGP1 and is required for its functional activity^40^. While increased Zn and AZGP1 secretion into the serum have been found in different human epithelial malignancies, we found for the first time that both Zn and AZGP1 levels were increased in the cytoplasmic compartment of HPV-positive OPSCC tumor cells. Further work is needed to elucidate the co-localization of Zn with AZGP1 in the cytoplasm of tumor cells.

Abundant evidence demonstrates that the loss of expression of AZGP1—just like Zn—in tumor cells is a negative prognostic biomarker for different solid tumors, including breast, gastric, esophageal, soft tissue and prostate cancers. In these cancers, loss of AZGP1 protein was also associated with an increased likelihood of lymph node metastasis and advanced clinical stage^26,41–45^. In the TME, data supports the conclusion that AZGP1 could function as a tumor suppressor gene through inhibition of ERK signaling^27^.

From our exploratory protein expression analysis by IHC, we found that a subset of all OPSCC patients overexpressed AZGP1 protein and had a significantly longer RFS compared to patients with absent or low AZGP1 expression, which we corroborated with survival analysis of HNSCC patients in the TCGA. In comparison to previous literature that reports increased AZGP1 staining in oral cavity squamous cell carcinoma with histologic evidence of maturation (keratinization), the vast majority (n = 12/14, 86%) of AZGP1-high OPSCC tumors in our cohort showed undifferentiated morphology (data not shown)^36^. This is in-keeping with the typical morphologic features of HPV-associated HNSCC and reflects an important distinction in AZGP1 staining patterns in distinct anatomic locations of the head and neck.

In light of these protein expression results, we wanted to explore corresponding *AZGP1* mRNA levels in HPV-positive and HPV-negative OPSCC patients. Our analysis of TCGA and GEO RNA-seq data for AZGP1 mRNA levels revealed that total mean *AZGP1* mRNA transcrips were higher in HPV-negative OPSCC, though it is impossible to make conclusions from this based on the small patient size of TCGA HPV-negative OPSCC cases. If *AZGP1* mRNA levels are indeed higher in HPV-negative OPSCC, our findings of its reduced expression at the protein level in our HPV-negative OPSCC, albeit also small in size and difficult to permit general conclusions, could be due to AZGP1 post-translational and/or epigenetic silencing through histone deacetylase (HDAC) activity, which is an established epigenetic silencing mechanism of AZGP1in different human cancers^27,46^.

While our methods did not allow us to segregate between episomal and integrated HPV-status in our OPSS cohort, we show that TCGA/GEEO cases with integrated HPV had significantly higher *AZGP1* mRNA levels compared to those cases with episomal HPV. What these findings from RNA-seq mean for the protein expression, however, remains to be established. The HPV oncoproteins E6 and E7 encode Zn finger transcription activation domains comprised of cysteine residues that are required for their function^47–49^. Additionally, the HPV16 E5 protein blocks the negative regulation of Zn redistribution into host cell nucleoli through interactions with the Zn transporter complex made of proteins EVER1 and 2 and ZnT-1^50^, resulting in increased cell proliferation. Further investigation is warranted to determine if HPV-related proteins such as EVER2 contribute to our finding that Zn is elevated in HPV-positive tumor cells. We speculate other Zn-binding proteins that are not encoded by the HPV genome, such as *AZGP1*, also contribute to increased detectable Zn levels in OPSCC cases.

This study is intended to draw previously unrecognized correlations between HPV-status in OPSCC with intratumoral trace element content and the expression of metal-binding proteins and not to establish mechanisms for which one finding may be related to another. We believe that both Zn and AZGP1 are promising therapeutic targets in HNSCC, though their role in patient sensitivity to chemoradiation needs to be further studied in a larger population. Major limitations of this study include the relatively small cohort size and the robustness of the correlation of overall tumor Zn levels with specific Zn binding proteins. With respect to the former, the number of patients in this cohort is not of adequate size to correlate to the general population. We attempted to remedy this by extending our survival analysis of AZGP1 to the larger HNSCC population database available in TCGA. Also, due to lack of tissue in some cases, we were not able to achieve statistical significance for a correlation between AZGP1 expression and Zn levels on a per-case basis.

## Methods

### Specimens

All archived patient specimens for retrospective analysis were collected from patients diagnosed with OPSCC at Northwestern Memorial Hospital (NMH), Department of Pathology under a Northwestern University (NU) IRB-approved protocol. All experiments were performed in accordance with relevant guidelines and regulations. The need for informed consent was waived by the NU IRB. Cases were selected over a six-year period (2010–2016) based on the following criteria: a primary diagnosis of squamous cell carcinoma of the oropharynx with available p16 immunohistochemistry (IHC) results and adequate tissue for testing. Among all patients, 16 HPV-positive and HPV-negative patient pairs were created (A.R.) for XFM imaging using the following matching variables: age, smoking, alcohol use, tumor differentiation, tumor location, tumor stage and gender.

FFPE tissues selected for this study were used for construction of TMAs as three 1.5 mm cores per case, comprised of two tumor cores and one core of adjacent histologically normal squamous mucosa, verified by a pathologist (K.P.). Tonsil cores from healthy donors were included in selection for the TMA as staining controls. Five μm sections were cut from tissue and TMA blocks for staining with hematoxylin and eosin (H&E) and for IHC. Tissue sections for XFM were placed on Ultralene membranes (SPEX Sample Prep, LLC, 15 Liberty St., Metuchen, NJ 08840, USA).

### HPV detection

At the time of diagnostic work-up, all cases were stained for p16—the HPV surrogate marker^51^—and interpreted as positive when >70% strong tumor nuclear and cytoplasmic staining was present. We also performed high-risk (HR) HPV testing on all cases using DNA and mRNA *in situ* hybridization (ISH). DNA ISH: ZytoFast® HPV High-Risk (HR) Types Probe specific for oncogenic E6/E7 from 15 HPV types, Cat. T-1140-400 combined with the Zytofast PLUS Implementation Kit, Cat. T-1061-40). mRNA ISH: RNAscope 2.0 HD Detection Kit (Brown, ACD, Cat. 322300). RNAscope testing used a probe against 18 high-risk (HR) HPV genotypes E6/E7 mRNA (RNAscope Probe HPV-HR18, ACD, Cat. 312591). Slides were processed according to the manufacturer’s instructions; the bacterial gene DapB was used as a negative control and housekeeping gene POLR2A served as a positive control.

### XFM imaging

XFM was done (T.P.) on FFPE whole-sections of tumor from patients with OPSCC (n = 32, 16 HPV-pos, 16 HPV-neg; Supplemental Fig. 1) at the 8BM-B and 2ID-E beamlines at the Advanced Photon Source at Argonne National Laboratory as previously described^52,53^. At the 8BMB beamline, the beam was focused to a 30 μm spot (low resolution) and at the beamline 2-ID-E the beam was focused to a 0.5 μm spot (medium resolution). Spectra were collected with a SII Vortex ME4 4-element silicon drift detector (SII NanoTechnology USA, Northridge, CA). Tissue sections were scanned with step sizes equal or larger than beam spot sizes and dwell times of 100–500 milliseconds; their elemental content was quantified and mapped. Elemental concentrations were calibrated using thin film AXO standards (Applied X-ray Optics, Dresden, Germany) and the data were calibrated using MAPS software^54^. Per pixel counts were converted to elemental concentrations (μg/cm^2^).

Data for 16 elements were collected: Zn, Mn, Fe, Cu, Co, phosphorous (P), sulfur (S), chlorine (Cl), potassium (K), calcium (Ca), chromium (Cr), nickel (Ni), arsenic (As), selenium (Se), bromine (Br) and mercury (Hg). Concentrations of some of the elements (Cr, Mn, Co, Ni, As, Se, Br, Hg) fell within the error range and those elements were not used for final analysis. Final measurement of elemental concentrations was done as follows: for each selected region of interest (ROI), mean per pixel value background area concentration for each element was subtracted from elemental concentrations for tumor and adjacent histologically benign squamous mucosa. Finally, these values were normalized relative to sulfur. Sulfur was chosen for this purpose due to the fact that it has a uniform signal in proteins from FFPE tissue, serving as a proxy for tissue density^55^. Normalizing to sulfur was also necessary because cell density and per cell sizes in ROI varied.

### Laser capture microdissection and mass spectroscopy

For two patients, FFPE tumor sections eight μm in thickness were mounted on membrane slides (Zeiss, Cat. 1.0 PEN 415190-9041) and were deparaffinized and stained for 3–5 seconds in 0.1% Toluidine Blue Solution (SIGMA, #T-0394). Laser capture microdissection (LCM) was performed using a type P-MB device (Carl Zeiss MicroImaging). From each section, areas of approximately 500,000 μm^2^ (roughly 100,000 cells) covering tumor were collected in adhesive caps 200 (Zeiss, Cat. 415190-9181-000). Tissue fragments were stored at −80 °C until use. Protein digestion with trypsin was done, followed by purification with C18 spin columns prior to analysis by mass spectrometry. Mass spectrometry data acquisition was done at NU Proteomics core facility. In brief, peptides were analyzed by LC-MS/MS using a Dionex UltiMate 3000 Rapid Separation nanoLC and a Q Exactive™ HF Hybrid Quadrupole-Orbitrap™ Mass Spectrometer (Thermo Fisher Scientific Inc, San Jose, CA). Peptides were separated on a 120-min analytical gradient from 5% ACN/0.1% FA to 30% ACN/0.1% FA. The top 15 most abundant precursor ions in each MS1 scan were selected for fragmentation. Proteins were identified from the tandem mass spectra extracted by Xcalibur version 4.0. MS/MS spectra were searched against the SwissProt Homo sapiens database using Mascot search engine (Matrix Science, London, UK; version 2.5.1). The search result was visualized by Scaffold (Proteome Software, INC., Portland, OR).

### Immunohistochemistry (IHC)

TMAs were cut at 5-um thickness and deparaffinized. Antigen retrieval was performed at 95 °C for 30 minutes followed by blocking with HRP solution. Slides were then incubated with antibodies for: AZGP1 (Atlas Antibodies, HPA012582, 1:500), Lipocalin-1 (Elabscience, E-AB-33489, 1:200), Albumin (Abcam, EPSISR1, 1:5,000), S100A7 (Novus Biologicals, NBP1-87205, 1:100), S100A8 (Mybiosource, CF-145, 1:400) and S100A9 (Cell Signaling Technology, D5O6O, 1:150) followed by HRP-conjugated secondary antibody and DAB staining. IHC staining in tumor for AZGP1 (cytoplasmic), Lipocalin-1 (cytoplasmic), Albumin (cytoplasmic), S100A7 (cytoplasmic and nuclear), S100A8 (cytoplasmic and nuclear) and S100A9 (cytoplasmic) were scored by a pathologist (KP) (Supplemental Fig. 1) on a scale from one to three, with one being the weakest staining and three being the strongest staining. All slides were digitized using Nanozoomer 2.0 (Hamamatsu).

### TCGA data extraction

We evaluated HNSCC patient clinical and RNA-seq data extracted from TCGA Research Network (http://cancergenome.nih.gov/) and the GEO (https://www.ncbi.nlm.nih.gov/geo/). The TCGA cohort was comprised of 515 patients with clinical and genomic data. For some analysis, RNA-seq data was extracted from HNSCC patients from the following coded sites: ‘oropharynx,’ ‘tonsil’ and ‘base of tongue’. Preprocessed and normalized htseq count data for *AZGP1* and *LCN1* gene expression was performed by aligning reads with STAR and Tophat2^28,56^. Detection of HPV status and integration sites was performed by realigning data to the HPV genome as previously described^28,57^. *AZGP1* RNAseq data for integrated and episomal HPV-positive OPSCC cases were obtained from: https://www.ncbi.nlm.nih.gov/geo/query/acc.cgi?acc=GSE74956. All downloaded data was subsequently normalized using the DESeq2R package^58^.

### Statistical methods

Comparisons between elemental concentrations were calculated using paired T tests. The relationship between HPV status and AZGP1/Lipocalin-1/Albumin/S100A7/S100A8/ S100A9 IHC scores were determined by linear regression and Pearson correlation. The relationship between log_2_-transformed *AZGP1* mRNA counts and HPV status was performed by Wilcoxon rank test. The Kaplan-Meier method was used for survival outcomes. The primary endpoint in survival outcomes for cases from NMH was post-treatment recurrence-free survival (RFS) starting at the date of diagnosis and defined as the absence of locoregional or distant recurrence or disease-related death. In HNSCC TCGA survival analysis, the primary endpoint was overall survival. Patient survival trends were tested by log-rank. Multivariable parametric a AFT models were performed to estimate associations between protein expression levels and survival outcomes. Covariate adjustments included: smoking status, age, sex and treatment with surgery only, radiation only, chemoradiation only, surgery followed by radiation or surgery followed by chemoradiation. *P* values ≤ 0.05 were considered statistically significant. All statistical analyses were performed in Prism (GraphPad Software) and R (v3.4).

## Supplementary information

Supplemental Information

## Data Availability

The datasets generated during and/or analysed during the current study are available from the corresponding author on reasonable request.
