# Supplementary material for: Elemental Zn and its Binding Protein Zinc-α2-Glycoprotein are Elevated in HPV-Positive Oropharyngeal Squamous Cell Carcinoma
Source: Sci Rep. 2019 Nov 18;9:16965. doi: 10.1038/s41598-019-53268-1 (PMC6861298; doi:10.1038/s41598-019-53268-1)
Supplement: Supplementary file 1 — Supplemental Information [file 41598_2019_53268_MOESM1_ESM.pdf]

Supplemental Information

Elemental Zn and its Binding Protein Zinc-α2-Glycoprotein are Elevated in HPV-Positive Oropharyngeal Squamous Cell Carcinoma

Kate Poropatich, Tatjana Paunesku, Alia Zander, Brian Wray, Matthew Schipma, Prarthana Dalal[1], Mark Agulnik MD, Si Chen, Barry Lai, Olga Antipova, Evan Maxey, Koshonna Brown, Michael Beau Wanzer, Demirkan Gursel, Hanli Fan, Alfred Rademaker, Gayle E Woloschak, Bharat B Mittal.

Supplemental Table 1: Clinicopathologic characteristics of patient cohort

	All cases		HPV-positive cases		HPV-negative cases		*P* Value[a]
**No. Cases**	**75**		**48**		**27**		
**Age at diagnosis, years (median)**	**58**		**58**		**59**		NS
	**n**	**(%)**	**n**	**(%)**	**n**	**(%)**	
**Sex**							
Male	**64**	(85.33)	**44**	(91.67)	**20**	(74.07)	NS
Female	**11**	(14.67)	**4**	(8.70)	**7**	(25.93)	NS
**Follow-up, years (mean)**[b]	3.28		3.71		3.38		NS
**Tobacco use**	**n= 70**		**n=44**		**n= 26**		
Never	25	(35.71)	19	(43.18)	6	(23.08)	NS
1-10 ppy	15	(21.43)	12	(27.27)	3	(11.54)	NS
10-20 ppy	13	(18.57)	5	(11.36)	8	(30.77)	NS
>20 ppy	17	(24.29)	8	(18.18)	9	(34.62)	NS
**T stage**	**n= 71**[c]		**n= 46**		**n= 25**		
1-2	57	(80.28)	41	(88.64)	16	(64.0)	**0.011**
3-4	14	(19.72)	5	(11.36)	9	(34.0)	
**N stage**	**n= 73**		**n= 46**		**n= 27**		
0-1	26	(35.62)	16	(36.36)	10	(37.04)	NS
2-3	47	(64.38)	28	(63.64)	17	(62.96)	
**Treatment**	**n= 72**		**n= 46**		**n= 26**		
Surgery	12	(17.91)	7	(15.22)	5	(19.23)	NS
Surgery + radiation	11	(16.42)	8	(17.39)	3	(11.54)	NS
Surgery + chemoradiation	35	(49.25)	24	(52.17)	11	(42.31)	NS
Chemoradiation	10	(11.94)	4	(8.70)	6	(23.08)	NS
Radiation	4	(4.48)	3	(6.52)	1	(3.85)	NS
**Recurrences**	17/73	(23.29)	7/46	(15.22)	10/27	(37.04)	**0.034**
**RFS, years (mean)**	3.20		3.71		2.51		**0.001**[d]
**Dead from disease**	7/73	(8.82)	3/46	(6.52)	4/27	(14.81)	NS

Abbreviations: RFS = Relapse Free Survival, ppy= pack per year, NS = Not Significant

[a]Unpaired Student's T test comparing HPV-positive and negative cases

[b]A subset of cases had available clinical follow-up (n= 73), smoking history (n= 70), treatment (n= 72), T staging (n= 71) and N staging (n= 73) data available in patient records.

[c] Two cases of unknown primaries

[d]Chi-squared test and univariate accelerated failure time (AFT) model

Supplemental Table 2: Results from Medium Resolution Scans for an HPV-positive and HPV-negative OPSCC patient pair

	Mean per pixel	Picogram	Std. Dev.
P (μg/cm^2) HPVpos	2.3197623	107.272	2.015699
HPVneg	1.893839	75.995	1.923522
K (μg/cm^2) HPVpos	0.20308988	9.391	0.199885
HPVneg	0.082197	3.298	0.122663
Ca (μg/cm^2) HPVpos	0.062696345	2.899	0.090326
HPVneg	0.088798	3.563	0.111999
Fe (μg/cm^2) HPVpos	0.022868192	1.057	0.028783
HPVneg	0.072591	2.912	0.056992
Cu (μg/cm^2) HPVpos	0.022817553	1.055	0.021368
HPVneg	0.015691	0.630	0.017686
Zn (μg/cm^2) HPVpos	0.036113113	1.670	0.027443
HPVneg	0.022473	0.902	0.020781

P= Phosphorous
S= Sulfur
K= Potassium
Fe= Iron
Cu= Copper
Zn= Zinc

Supplemental Table 3: Proteomics results

Protein	HPV-Positive Patient	HPV-Negative Patient	Both patients	Zn-Binding
LCN1	×			×
KRT15	×			
ATP1A1	×			
LACRT	×			
HNRNPU	×			
KRT8	×			
HSPD1	×			
DHX9	×			
CSTB	×			
DDX17	×			
DSG3	×			
PCNA	×			
GOT2	×			
CPNE3	×			
RPL5	×			
SET	×			
RPLP0	×			
CCT8	×			
MDH2	×			
RPS21	×			
PDIA4	×			
SERPINB13	×			
YWHAQ	×			
GSTP1	×			
CSTA	×			
RRBP1	×			
WFDC12	×			
RNH1	×			
IGKV3-15	×			
CASP14	×			
SERPINB5	×			
LY6D	×			
KPRP	×			
SERPINB12	×			
ARG1	×			
SH3BGRL	×			
SLPI	×			
TGM3	×			
DBI	×			
PRDX4	×			
PSAP	×			

LYPD3	×			
MZB1	×			
EPPK1	×			
PI3	×			
RPLP2	×			
FLG	×			
CA1		×		×
COL18A1		×		×
HLA-DRB1		×		
C4A		×		
CFB		×		
APOB		×		
AKR1C3		×		
ITIH4		×		
GC		×		
AZU1		×		
DPYSL2		×		
COL4A1		×		
MBP		×		
PRX		×		
LAMB2		×		
NEFL		×		
PLG		×		
VTN		×		
F13A1		×		
DCN		×		
ELANE		×		
COL15A1		×		
CD163		×		
F2		×		
EMILIN1		×		
A1BG		×		
LAMC1		×		
FTL		×		
C9		×		
APOD		×		
NEFH		×		
OGN		×		
FTH1		×		
PRELP		×		
PRPH		×		
MPZ		×		
AZGP1			×	×
ALB			×	×
S100A7			×	×
S100A8			×	×

S100A9			×	×
UBA52			×	×
NCL			×	
KRT13			×	
PLEC			×	
A2M			×	
CFH			×	
COL4A2			×	
TGM2			×	
GSN			×	
TTR			×	
CALM1			×	
ANXA6			×	
CAP1			×	
IGHV4-34			×	
CORO1A			×	
HLA-B			×	
THBS1			×	
TXNDC5			×	
VCAN			×	
S100A6			×	
COL1A2			×	
EFEMP1			×	
HPX			×	
COL1A1			×	
TAGLN			×	
YWHAE			×	
COL14A1			×	
CKAP4			×	
APCS			×	
CP			×	
FBLN1			×	
ORM1			×	
LGALS1			×	
LUM			×	
IGHM			×	
CLU			×	
FLNA			×	
POSTN			×	
BGN			×	
COL12A1			×	
SERPINA1			×	
APOA1			×	
FGA			×	
TNC			×	
HSPG2			×	

HP			×	
COL6A2			×	
COL6A1			×	
FGG			×	
C3			×	
FBN1			×	
COL6A3			×	
RPS11			×	
TUBB4B			×	
PGD			×	
TAGLN2			×	
HSP90AA1			×	
PCBP1			×	
RACK1			×	
RPL6			×	
RPS18			×	
EIF4A1			×	
RPS4X			×	
HNRNPD			×	
PGK1			×	
PGAM1			×	
RPS14			×	
PRDX6			×	
RPSA			×	
PFN1			×	
P4HB			×	
ANXA1			×	
MSN			×	
HNRNPK			×	
FBLN2			×	
SFN			×	
TYMP			×	
FGB			×	
FN1			×	
TF			×	
IGHA1			×	
LTF			×	
HSP90B1			×	
EEF1G			×	
CTSG			×	
IGKV2-28			×	
CFL1			×	
SERPINH1			×	
ACTN1			×	
PDIA3			×	
LYZ			×	

RPL8			×	
SOD2			×	
IGKV3-20			×	
KRT19			×	
MPO			×	
TGFBI			×	
IGHG2			×	
IGHG3			×	
DSG1			×	
TPM4			×	
SERPINA3			×	
PKP1			×	
CALML3			×	
HSPA1A			×	
HSPA1A			×	
PIP			×	
KRT6C			×	
KRT6B			×	
TKT			×	
CBR1			×	
RPL7			×	
PTMA			×	
S100A11			×	
RPL12			×	
HIST1H1D			×	
RPL13			×	
HNRNPC			×	
MIF			×	
RPL22			×	
RPL4			×	
EEF2			×	
RPL18			×	
RPS2			×	
ALDOA			×	
HSP90AB1			×	
MYL6			×	
HSPA5			×	
MYH9			×	
HNRNPA2B1			×	
HRNR			×	
RPS3			×	
EEF1A1P5			×	
ENO1			×	
LMNA			×	
TUBB			×	
VIM			×	

FLG2			×	
PRDX2			×	
HSPB1			×	
LDHA			×	
YWHAZ			×	
HIST1H1B			×	
PPIA			×	
HBA1			×	
CALML5			×	
HSPA8			×	
TPI1			×	
ATP5B			×	
HIST1H4A			×	
H3F3A			×	
TUBA1B			×	
HIST2H2AA3			×	
DEFA3			×	
FABP5			×	
DSP			×	
ATP5A1			×	
PKM			×	
PRSS1			×	
HBB			×	
PRDX1			×	
JUP			×	
ANXA2			×	
HIST1H2BC			×	
ACTC1			×	
GAPDH			×	
ACTB			×	
DCD			×	
KRT17			×	
KRT14			×	
KRT5			×	
KRT16			×	
KRT6A			×	
KRT2			×	
KRT9			×	
KRT10			×	
KRT1			×	
HNRNPA1			×	
RPL7A			×	
TALDO1			×	
LGALS7			×	
KRT78			×	
SBSN			×	

DSC1			×	
CDSN			×	
P0DOX2			×	
P0DP06			×	
P0DP08			×	

Supplemental Table 4: Immunohistochemistry results for Zn-binding Proteins

	All Cases				HPV-positive cases				HPV-negative cases				P^a
	Tumor Tissue (n)		Normal Tissue (n)		Tumor Tissue (n)		Normal Tissue (n)		Tumor Tissue (n)		Normal Tissue (n)		
**AZGP1**	**68**	**(%)**	**33**	**(%)**	**45**	**(%)**	**22**	**(%)**	**23**	**(%)**	**11**	**(%)**	**0.0001**
Low	15	22	17	52	5	11	13	59	10	44	3	27	
Medium	39	57	13	39	27	60	7	23	12	52	7	64	
High	14	21	3	9	13	29	2	9	1	4	1	9	
Median	2.0		1.5		2.0		1.5		1.5		1.75		
**Lipocalin-1**	**63**	**(%)**	**34**	**(%)**	**41**	**(%)**	**19**	**(%)**	**22**	**(%)**	**15**	**(%)**	NS
Low	8	13	7	21	6	15	5	26	2	9	2	13	
Medium	38	60	12	35	26	63	8	38	12	55	4	27	
High	17	27	15	44	9	22	6	29	8	36	9	60	
Median	2.25		2.0		2.0		2.0		2.5		2.5		
**Albumin**	**26**	**(%)**	**15**	**(%)**	**21**	**(%)**	**12**	**(%)**	**5**	**(%)**	**3**	**(%)**	NS
Low	16	62	7	47	13	63	6	50	3	60	1	33	
Medium	6	23	1	6	6	29	1	8	0	0	0	0	
High	4	15	7	47	2	10	5	42	2	40	2	67	
Median	1.25		1.75		1.5		1.5		0.25		2		
**S100A7**	**53**	**(%)**	**26**	**(%)**	**43**	**(%)**	**21**	**(%)**	**10**	**(%)**	**5**	**(%)**	NS
Low	33	62	14	54	23	53	13	62	4	40	2	40	
Medium	12	23	3	12	12	28	1	5	4	40	2	40	
High	8	15	9	34	8	19	7	33	2	20	1	20	
Median	1		1		1		1		2		2		
**S100A8**	**51**	**(%)**	**26**	**(%)**	**41**	**(%)**	**21**	**(%)**	**10**	**(%)**	**4**	**(%)**	NS
Low	19	37	0	0	9	22	0	0	2	20	0	0	
Medium	7	14	8	31	11	27	7	33	3	30	0	0	
High	25	49	18	69	21	51	14	67	5	50	4	0	
Median	2.5		3		2.5		3		2.25		3		
**S100A9**	**50**	**(%)**	**26**	**(%)**	**41**	**(%)**	**21**	**(%)**	**9**	**(%)**	**5**	**(%)**	NS
Low	18	36	1	4	13	32	1	2	5	56	0	0	
Medium	10	22	10	38	8	20	10	48	2	22	0	0	
High	22	44	15	58	20	49	10	48	2	22	5	100	
Median	2.5		2		2		2.25		1.75		3		

Abbreviations: NS = not significant.

[a]Reported for tumor Pearson correlation between HPV-positive and negative patients.

Supplemental Figure 1

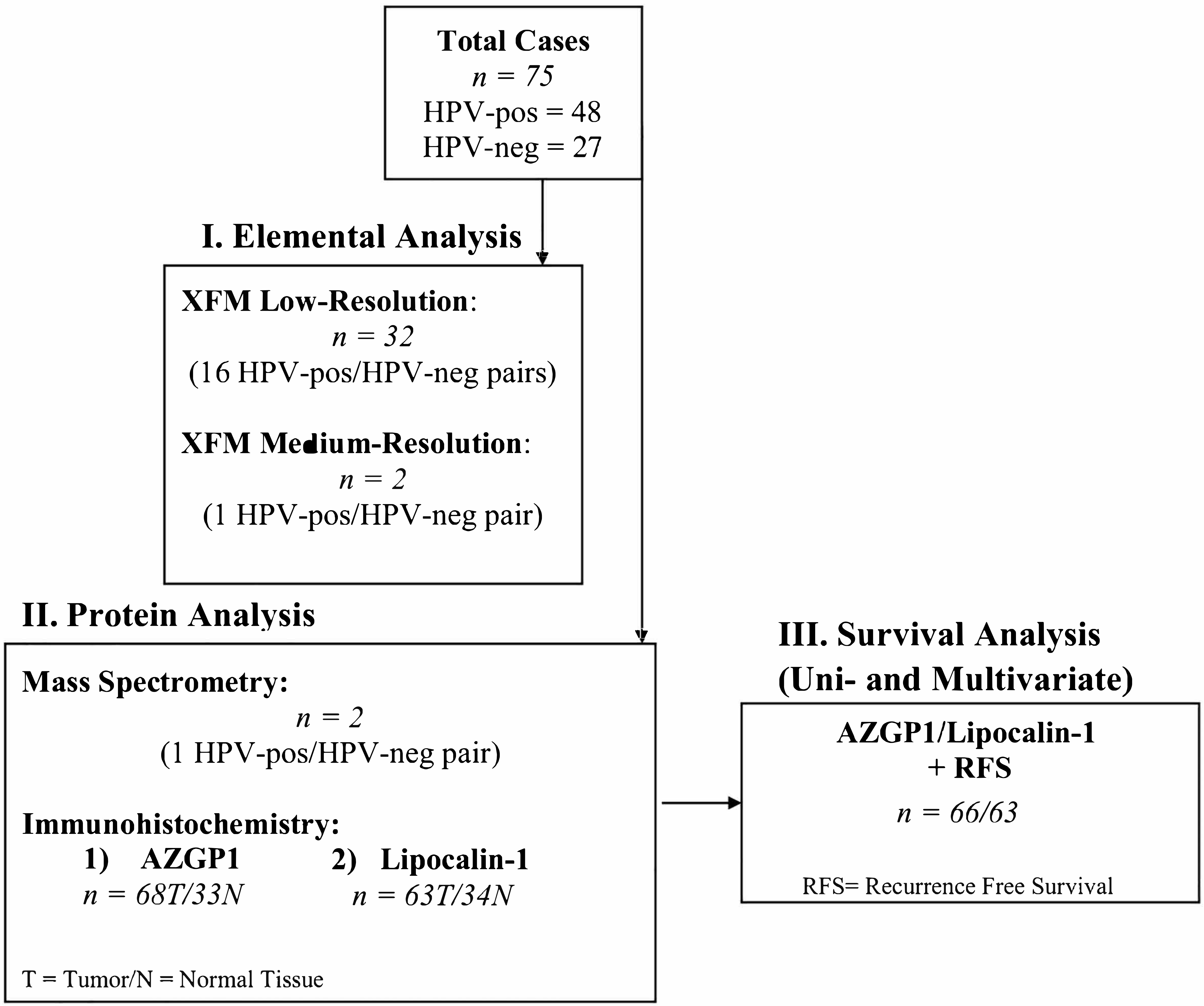

Supplemental Figure 2

Pair 1 ROI: 40 tumor cells

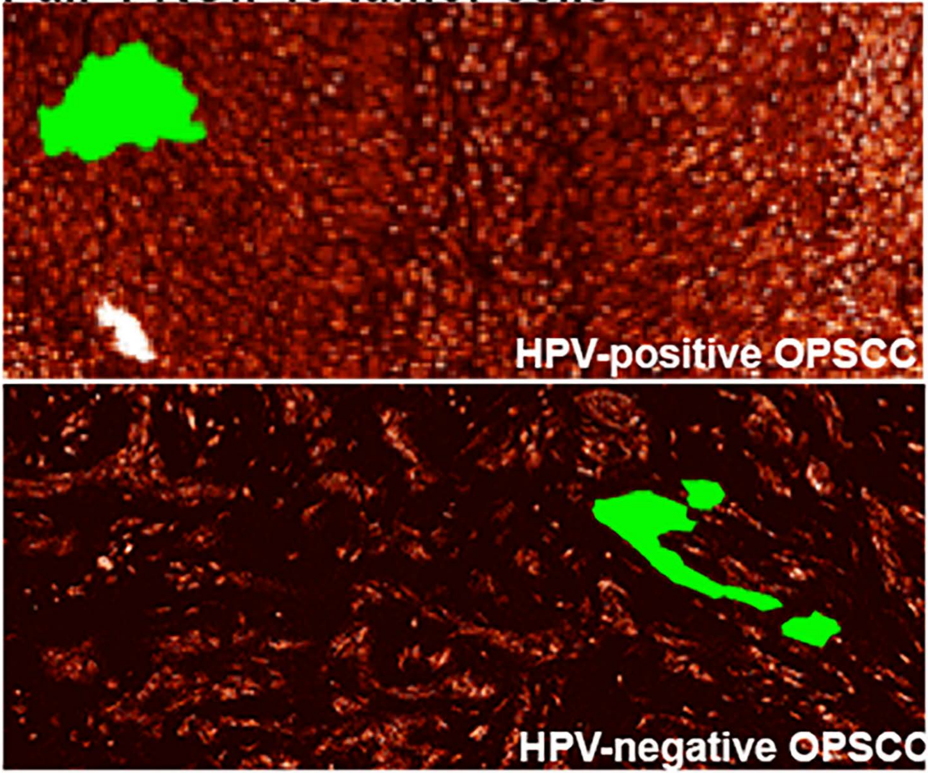

Supplemental Figure Legends

Supplemental Figure 1

Study design flow schemata: A total of 75 cases of OPSCC at Northwestern Memorial Hospital over a six year period were identified with sufficient tissue for further testing, of which a matched HPV-positive and HPV-negative subset underwent elemental analysis by XFM (n= 32) and mass spectrometry (n= 2). Based on results from these assays, two proteins—AZGP1 and Lipocalin-1—were selected for further measurement in OPSCC by immunohistochemistry (IHC) and subsequently correlated to multivariate Kaplan-Meier survival analysis (log-rank).

Supplemental Figure 2

Example of medium-resolution XFM analysis for elemental quantification in region of interests (ROI) drawn over 40 tumor cells, excluding surrounding necrosis, neo-vascularization and/or other sources of potential contamination.
